# The Transdiagnostic Intervention for Sleep and Circadian Dysfunction (TSC) in Community Mental Health: Evaluating Self-Reported Psychiatric Disorders as a Predictor of Symptoms and Treatment Outcome

**DOI:** 10.21203/rs.3.rs-7189279/v1

**Published:** 2025-08-06

**Authors:** Tanya B. Horwitz, Laurel D. Sarfan, Anne E. Milner, Joshua Varghese, Catherine A. Callaway, Allison G. Harvey

**Affiliations:** University of California, Berkeley; Kaiser Permanente; University of California, Berkeley; University of California, Berkeley; University of California, Berkeley; University of California, Berkeley

## Abstract

**Objective:**

This study investigated the extent to which self-reported psychiatric disorders and comorbidities in patients receiving the Transdiagnostic Intervention for Sleep and Circadian Dysfunction (TSC) predicted symptom/impairment severity and treatment outcome.

**Method:**

This secondary analysis drew from a subset of 489 patients in California-based Community Mental Health Centers with serious mental illness (SMI) and sleep/circadian problems who had participated in a randomized controlled trial of TSC. Of these patients, 253 received Immediate TSC and 236 received usual care followed by delayed treatment (UC-DT). Some patients received Standard TSC (N=149) while others received Adapted TSC (N=340). We analyzed patients’ baseline/pre-treatment and post-treatment scores for psychiatric symptoms, sleep disturbance, sleep-related impairment, overall sleep health, and functional impairment. We also used patient-reported data on history of psychiatric diagnoses.

**Results:**

At baseline, patients with more disorders had worse psychiatric symptom scores (b=2.27, SE=0.39, p<.001), greater sleep disturbance prior to eliminating outliers (b=0.71, SE=0.31, p=.023), greater sleep-related impairment (b=1.25, SE=0.34, p<.001), worse overall sleep health (b=−0.20, SE=0.06, p=.001), and greater functional impairment (b=0.32, SE=0.11, p=.005). Number of psychiatric disorders was not associated with treatment outcome or differences in benefits between Standard and Adapted TSC. When analyzed independently, the most common psychiatric disorder groups all demonstrated significantly more improvement in the Immediate TSC group than in the UC-DT group on sleep disturbance and sleep-related impairment.

**Conclusions:**

These results build on strong evidence that transdiagnostic sleep treatment is an important approach to treating populations with comorbid SMI and sleep/circadian problems. This secondary analysis was not pre-registered but uses data from primary analyses that were pre-registered on clinicaltrials.gov (phase 1/generation 1: https://clinicaltrials.gov/study/NCT04154631; phase 2/generation 2: https://clinicaltrials.gov/study/NCT05805657).

## INTRODUCTION

About 50% of people with mental illness have two or more psychiatric disorders (Andrews et al., 2002; Bijl et al., 1998; Kessler et al., 2005). Psychiatric disorders are highly comorbid with sleep and circadian problems, which are highly comorbid with one another (Freeman et al., 2020; Harvey et al., 2003; Roth et al., 2006; Rumble et al., 2015). Moreover, the diagnostic criteria for multiple psychiatric disorders—including depressive and bipolar spectrum disorders, multiple anxiety and trauma and stressor-related disorders, and substance use withdrawal—explicitly include symptoms relating to sleep and circadian problems (Harvey, 2008). Additionally, sleep and circadian problems have been linked to the development and course of psychiatric disorders (Chemerinski et al., 2002; Freeman et al., 2020; Harvey, 2008; Rumble et al., 2015; Sivertsen et al., 2012). The co-occurrence of sleep/circadian and other psychiatric problems may be due to bidirectional processes, common etiological connections (i.e., shared genetic and genomic causes (Morrison et al., 2023)), and/or relationships among dynamic cognitive, emotional, and biological processes. For example, sleep loss has been linked to decreased activation of and decreased functional connectivity in brain regions associated with executive function, which could in turn augment anxiety symptoms via decreased inhibition of worry, rumination, and other associated processes (Cox & Olatunji, 2016). Moreover, it is possible that sleep/circadian problems and psychiatric symptoms exacerbate each other in a “feedback loop”. For example, an individual with a sleep or circadian problem may use substances in an attempt to improve their sleep and use stimulants to cope with fatigue during the day, heightening the risk of substance use disorder and potentially exacerbating the individual’s original sleep problem (American Psychiatric Association, 2022).

Research on psychiatric comorbidity and multimorbidity^1^ points to several prognostic, social, and clinical implications. For instance, the National Comorbidity Survey Replication, a nationally representative household survey of adult English speakers in the United States, reported that 49.9% of respondents with three or more psychiatric disorders—as compared to 25.5% with two disorders and 9.6% with one disorder—qualified as serious cases. Here, serious cases were defined according to factors relating to suicide attempts, role impairment, violence, psychosis and bipolar status, and/or serious substance use disorder (Kessler et al., 2005). Also, a cohort study following a sample of adults in Zurich, Switzerland found that a greater number of psychiatric disorders was associated with greater subjective distress and work impairment, poorer quality of life, and higher current symptom scores (Angst et al., 2002).

The treatment implications of psychiatric comorbidity are more complex. While some treatment studies list comorbidity in their exclusion criteria, other studies’ findings are difficult to generalize because they typically focus on comorbidities related to specific index conditions. For example, one 2010 meta-analysis studied the relationship between comorbidity in anxiety disorders and treatment outcome, yielding mixed results (Olatunji et al., 2010). There was no overall relationship between the samples’ percentage of comorbid patients (those with at least one additional disorder) and treatment effect at post-treatment when collapsing across all anxiety disorders. However, the direction and significance of the relationship between comorbidity and treatment effect at post-treatment varied across *specific* anxiety subgroups (i.e., social phobia). That is, depending on the subgroup, having at least one comorbid condition could be a positive, negative, or non-significant predictor of treatment effect at post-treatment. Additionally, at *follow-up*, a significant relationship between comorbidity and treatment effect remained for only one subgroup. More research is needed to better understand comorbidities across the full psychiatric spectrum and to understand the effectiveness of treatments that target a range of psychiatric diagnoses and comorbidities.

Transdiagnostic treatments aim to treat processes that are common to comorbid disorders and problems (Fairburn et al., 2003; Harvey, 2004). The present study is a secondary analysis of patients diagnosed with serious mental illness (SMI) who were treated with the Transdiagnostic Intervention for Sleep and Circadian Dysfunction (TSC) (Harvey et al., 2025) by providers in Community Mental Health Centers (CMHCs). Because a range of sleep and circadian problems are common in people who have been diagnosed with SMI, TSC is intended to address hypothesized shared “underlying” mechanisms in patients with a diverse range of psychiatric presentations and comorbidities (Callaway et al., 2023; Harvey et al., 2021, 2025; Harvey & Buysse, 2018; Sarfan et al., 2023). In addition to alleviating sleep- and circadian- related problems, TSC has been associated with general improvement in psychiatric symptoms (Harvey et al., 2021, 2025; Yau et al., 2024) and functional impairment (Harvey et al., 2021, 2025), among other outcomes.

The goal of the current study is to characterize how the psychiatric disorders reported by patients relate to baseline measures of sleep, impairment, and psychiatric outcomes as well as change in the outcomes following participation in a course of TSC. We focus here on non-sleep and circadian disorders because sleep and circadian disorders were not systematically collected for the current sample and because our team has focused on sleep and circadian problems in prior research. In particular, Sarfan et al. (2021) found that after adjusting for diagnostic threshold (i.e., whether disorders met full diagnostic criteria) in a community sample receiving TSC, patients with more *sleep and circadian* problems had less favorable scores at baseline for most outcomes (Sarfan et al., 2021). Meanwhile, Sarfan *et al*. did not find evidence that benefits of TSC—as measured by patients’ improvement on outcomes over the course of treatment—varied based on number of sleep and circadian problems. However, this may be because TSC focuses on sleep and circadian problems (as opposed to other types of psychiatric symptoms). Thus, it is possible that the results (i.e., baseline scores and TSC outcomes) would be different if the number of *psychiatric disorders* is the variable of interest. Additionally, the sample analyzed in Sarfan et al. (2021) received TSC from UC Berkeley providers; it is possible that outcomes would differ for patients in the present sample because the latter received treatment from “real world” providers.

The context for data collection for this study is noteworthy: Community Mental Health Centers (CMHCs) (and Community Health Centers more broadly) are a primary resource for patients who are of low socioeconomic status (Lee et al., 2024), making CMHCs an important context for testing and implementing evidence-based interventions (EBIs). Unfortunately, CMHCs are often under-resourced (Harvey et al., 2025), which has negative implications for optimizing provider training and supervision (Peterson et al., 2019). These factors must be considered in conjunction with general challenges of EBI implementation such as clinician resistance to change (Lilienfeld et al., 2013) and low penetration rates (Bruns et al., 2016). In light of these considerations, an “Adapted” version of TSC has also been developed to enhance feasibility in CMHC settings (Callaway et al., 2023; Harvey et al., 2025; Sarfan et al., 2023), as described below.

The patients in the present community sample were assigned to receive either Standard or Adapted TSC based on the county of their CMHC. The latter version of TSC is briefer and was designed based on feedback, theory, and pilot data to improve the “fit” of TSC to the CMHC context (Sarfan et al., 2023). Interestingly, in the first phase/generation of the current paper’s parent trial––during which patients received TSC from providers at CMHCs who were trained by University of California (UC) Berkeley providers (Sarfan et al., 2023)––CMHC providers’ ratings of fit did not differ for Adapted versus Standard TSC after accounting for pre-treatment (Harvey et al., 2025). Additionally, fit did not mediate the relationship between version of the treatment (Adapted vs. Standard) and patient outcomes for the main measures (Harvey et al., 2025). Feedback from CMHC providers who delivered TSC has suggested that there are several advantages of Adapted over Standard, although there may be a few ways in which Standard is preferable to Adapted. In a qualitative analysis of interviews with CMHC TSC providers, Adapted providers reported more favorable views of treatment characteristics, and Standard providers were generally more concerned that TSC was not appropriate for all patient presentations. At the same time, more Adapted than Standard providers reported that they would require additional resources to continue delivering the treatment (Sarfan et al., 2024).

Building on this prior research, the current study will investigate whether the relative advantages of Adapted versus Standard TSC depend on the number of psychiatric disorders an individual has. This study will also compare patients who received TSC immediately (Immediate TSC) in addition to the usual care they were already receiving at their CMHC to patients who received usual care only, followed by delayed treatment with TSC (UC-DT). Of note, in the current study, psychiatric disorders and the number of psychiatric disorders were based on patients’ recall of their psychiatric diagnoses. The gold standard for psychiatric diagnosis involves trained professionals administering structured interviews. However, such methods can be long and thus burdensome to the patient, particularly in settings like CMHCs. Indeed, while some such data was collected, limited resources precluded a consistent, standardized provider-based diagnostic procedure across the entire sample. That said, a patient may have a better sense of their psychiatric history than their current provider does, especially in new patient-provider relationships. By leveraging patients’ reports of their own psychiatric diagnostic histories, the present study supports patient-centered perspectives as a valuable window into mental health challenges (Glas, 2022; Mezzich, 2007; Salloum & Mezzich, 2010).

Aim 1 of the present study will investigate the relationship between the number of psychiatric disorders and patients’ baseline scores on five outcomes: cross-cutting psychiatric symptoms, sleep disturbance, sleep-related impairment, overall sleep health, and overall functional impairment. We hypothesize that a higher number of psychiatric disorders was associated with more psychiatric symptoms, more sleep disturbance, more sleep-related impairment, poorer overall sleep health, and greater overall functional impairment at baseline. Aim 2 will assess if the number of psychiatric disorders was associated with differences in benefits between the Immediate TSC and the UC-DT conditions. We hypothesize that, across all outcomes, the benefits of TSC relative to UC-DT were greater for people with fewer psychiatric disorders. Aim 3 will assess if the number of psychiatric disorders was associated with differences in benefits between Standard and Adapted TSC. We expect that, owing to the longer and more comprehensive nature of Standard TSC, patients with more diagnoses benefited more from Standard TSC, relative to Adapted TSC. Aim 4 will test differences in improvement across Immediate TSC and UC-DT within each broad psychiatric disorder group (i.e., people with anxiety disorders) independently. As TSC was developed to treat sleep and circadian problems in patients with a wide range of SMIs, we hypothesize that TSC was associated with more improved outcomes, relative to usual care alone, for all psychiatric disorder groups.

## METHODS

### 2.1 Study Overview and Patients

The data for this study were drawn from a hybrid type 2 effectiveness-implementation study (Curran et al., 2012) conducted in the Community Mental Health Centers (CMHCs) of counties across California in the United States. The “parent” study was comprised of three phases, two of which are relevant here. In the first phase—which is referred to as “generation 1”—patients received TSC from providers at CMHCs who were trained by University of California (UC) Berkeley providers (Sarfan et al., 2023). In the second phase—which is referred to as “generation 2”—patients received treatment from CMHC providers trained by other CMHC providers (Callaway et al., 2023). The sample of patients (*N* = 539) was recruited from CMHCs in one of ten California counties: Alameda, Contra Costa, Kings, Monterey, Placer, Santa Cruz, Solano, Santa Clara, Santa Barbara (generation 1 only), and Lake (generation 1 only). Note that sites in San Luis Obispo also participated but were operating as part of Monterey County (Callaway et al., 2023).

Of the patients who were ultimately enrolled, 276 were randomized to receive Immediate TSC and 263 were randomized to usual care followed by delayed treatment (UC-DT). Within the full enrolled sample, 161 patients received Standard TSC, while 378 received Adapted TSC (described below).

The inclusion criteria for selecting the CMHC sites from which to recruit providers and patients were as follows: (1) provision of publicly funded adult mental health outpatient services and (2) support from CMHC leadership. CMHCs preferred to determine which providers (e.g., case managers, nurses, psychiatrists) were eligible to receive TSC training at each site.

As specified in the protocol papers (Callaway et al., 2023; Sarfan et al., 2023), all patients met the following inclusion criteria: (1) aged 18 years and older; (2) had serious mental illness (SMI), defined here as at least one *Diagnostic and Statistical Manual* (DSM) mental disorder causing substantial interference with one or more major life activities for at least 12 months (Wang et al., 2002). Data on SMIs were collected based on self-report. Some patients’ providers also reported patients’ diagnoses, and for some patients, the Mini International Neuropsychiatric Interview (DSM-5, Version 7.0.0) was administered by a licensed clinical social worker on the research team;^2^ (3) scored a 4 (“quite a bit”) or 5 (“very much”), or the equivalent for reverse scored items, on one or more PROMIS-Sleep Disturbance (PROMIS-SD) items (see Patient Outcome Measures); (4) had a guaranteed place to sleep for at least 2 months other than a shelter; (5) received the standard of care for the SMI and consented to regular communications between the research team and provider; and (6) consented to having their medical information accessed and to participating in assessments (Callaway et al., 2023; Harvey et al., 2025; Sarfan et al., 2023).

Patients were excluded if they met any of the following criteria: (1) presence of an active and progressive physical illness or neurological degenerative disease directly related to the onset and course of the sleep and circadian problems, or that made participation in the study unfeasible, as assessed by the Checklist of Medical Conditions and Symptoms on the Duke Structured Interview for Sleep Disorders (Edinger et al., 2004) and clinical interview; (2) presence of substance abuse/dependence, only if it made participation in the study unfeasible; (3) current active intent or plan to commit suicide (those with suicidal ideation were eligible) only if it made participation in the study unfeasible, or homicide risk; (4) night shift work for more than 2 nights per weeks in the past 3 months (i.e., regularly scheduled work from 12 a.m. – 6 a.m.); or (5) pregnant or breastfeeding (Callaway et al., 2023; Harvey et al., 2025; Sarfan et al., 2023).

### 2.2 Treatments

Two variations of TSC were tested: Standard TSC and Adapted TSC. Sites were cluster-randomized by county to Adapted TSC or Standard TSC with 1:1 allocation. Throughout the trial, all patients received usual care, which consisted of working with a service provider (e.g., psychologist, case manager, occupational therapist, psychiatrist, nurse practitioner) who provided mental health support in addition to other services, as needed (e.g., housing support). Patients in the UC-DT condition began with usual care for approximately^3^ 4 or 8 weeks, depending on whether their CMHC was randomized to Adapted TSC or Standard TSC, respectively. After the delay, they received Adapted or Standard TSC, depending on their CMHC. Supplementary Table 1 summarizes the treatment modules for the Standard and Adapted conditions.

Although most providers delivered TSC via individual sessions, some opted to deliver it in a group setting. TSC was originally developed in English, then translated into other languages to expand access. TSC was offered by Spanish-speaking providers to some Spanish-speaking patients (*N* = 25 providers, *N* = 42 patients) and by Vietnamese-speaking providers to some Vietnamese-speaking patients (*N* = 3 providers, *N* = 3 patients).

#### 2.2.1 Standard TSC

Standard TSC was designed to be delivered by CMHC providers across eight 50-minute, weekly sessions (Harvey & Buysse, 2018). It is comprised of four cross-cutting modules featured in every session, four core modules, and seven optional modules to be used based on clinical presentation, treatment goals, and provider case conceptualization. Training for the Standard TSC condition consisted of a 1-day workshop (i.e., 6–8 hours) or two 3-hour training blocks, depending on CMHC preference.

#### 2.2.2 Adapted TSC

Adapted TSC was designed to be delivered by CMHC staff across four 20-minute, weekly sessions. Treatment consists of the same four cross-cutting modules as in Standard TSC, all but one of the same core modules^4^, and one of the same optional modules. Training for the Adapted TSC condition consisted of four 1-hour workshops or two 2-hour workshops, depending on CMHC preference.

### 2.3 Measures

In addition to the measures below, a sociodemographics form was completed by providers and patients.

#### 2.3.1 Patient Outcome Measures

The following five measures were analyzed as the outcome variables (separately) across all aims:

**Psychiatric Symptoms** (distinct from Psychiatric Disorder Variables see below) were measured using the DSM-5 Level 1 Cross-Cutting Symptom Measure—Adult (American Psychiatric Association, 2013; Clarke & Kuhl, 2014; Narrow et al., 2013), a 23-item questionnaire that assesses how much patients were bothered by various symptoms over the past 2 weeks. Items on the questionnaire are grouped into 13 domains, with the highest item score within a domain used as the “domain score.” Raw scores range from 0–4 on each item such that possible total scores—summed across domains—range from 0–52, where higher scores indicate greater levels of distress relating to psychiatric symptoms. Because items are summed, only patients with all 13 domain scores on the measure were retained for DSM-5 Cross-Cutting analyses. This measure has demonstrated good test-retest reliability and clinical utility (Clarke & Kuhl, 2014; Narrow et al., 2013).

**Sleep Disturbance** was measured using patients’ standardized scores on the Patient-Reported Outcomes Measurement Information System-Sleep Disturbance (PROMIS-SD) 8b short form (Buysse et al., 2010; Yu et al., 2012), an 8-item questionnaire that assesses disruption to sleep (e.g., trouble staying asleep) over the past week. Raw scores range from 1–5 on each item such that possible raw total scores—summed across items—range from 8–40, where higher scores indicate greater levels of sleep disturbance. Because a conversion table requiring complete responses was used for standardization, only patients who responded to all eight items on the measure were retained for PROMIS-SD analyses. Possible standardized scores (used for analyses and results) range from 28.9 to 76.5 (see the appendix of Yu et al. (2012) (Yu et al., 2012) for details). This measure has demonstrated acceptable reliability and validity (Buysse et al., 2010; Yu et al., 2012).

**Sleep-Related Impairment** was measured using patients’ standardized scores on the Patient-Reported Outcomes Measurement Information System-Sleep Related Impairment (PROMIS-SRI) 8a short form (Buysse et al., 2010; Yu et al., 2012), an 8-item questionnaire that assesses daytime impairment related to sleep problems over the past week. Because the method for standardizing PROMIS-SRI scores was similar to that used for the PROMIS-SD and required complete responses, only patients who responded to all eight items on the measure were retained for PROMIS-SRI analyses. Raw scores range from 1–5 on each item such that possible raw total scores—summed across items—range from 8–40, where higher scores indicate greater levels of sleep related impairment. Possible standardized scores (used for analyses and results) range from 30 to 80.1 (see the appendix of Yu et al. (2012) (Yu et al., 2012) for details). This measure has demonstrated excellent psychometric properties (Buysse et al., 2010; Yu et al., 2012).

**Overall Sleep Health** was measured using the Sleep Health Composite (SHC) (Dong et al., 2019), a measure comprised of six dimensions: Regularity (midpoint fluctuation), Timing (mean midpoint), Efficiency (sleep efficiency), Duration (total sleep time), Satisfaction (sleep quality question on PROMISSD), and Alertness (daytime sleepiness question on PROMIS-SRI), each with binary response options (1 = good; 0 = poor). Each item except Satisfaction and Alertness pertains to patterns over the past 7 days. Possible total scores—summed across items—range from 0–6, where higher scores indicate better sleep health. Because items are summed, only patients who responded to all 6 items on the measure were retained for SHC analyses. Initial validity of this measure has been established (Dong et al., 2019).

**Functional Impairment** was measured using the Sheehan Disability Scale (SDS) (Sheehan et al., 1996). Items are rated on a scale from 1 (not at all) to 10 (extremely), with higher scores indicating greater impairment. The three items on the SDS are typically summed to achieve patients’ total scores. However, there was a markedly high rate of missingness on Item 1 (*N* = 282 of the original 539 enrolled patients had missing responses at baseline). Because the first item asks about problems at work or school, this was likely due to the high rate of unemployment in the current sample (only 32% of the 539 patients reported full- or part-time employment). In order to avoid downward bias in patients who did not give a response to Item 1, we instead used the mean value across the (1–3) non-missing SDS items for each patient. The SDS has demonstrated good reliability and validity (Leon et al., 1997; Sheehan et al., 1996).

#### 2.3.2 Psychiatric Disorder Variables

For the present analyses, patients’ psychiatric disorders were primarily determined based on their responses to the question: “*Have you been diagnosed with a mental health issue such as ADHD, autism spectrum disorder, anxiety, depression, bipolar disorder, OCD, PTSD, substance dependence, or schizophrenia*?” If the patient responded “yes,” they were asked: “*What is/are your diagnosis(es)?*” Two coders initially coded the patient responses with respect to psychiatric disorders in the DSM-5 or DSM-5-TR^5^ (for ease of communication, we will use “DSM-5” to refer to both going forward).

Patients could be coded for a disorder in one of two ways: either by listing a disorder that directly matched a DSM-5 entry (i.e., major depressive disorder) or by listing a vague or generic disorder (i.e., “anxiety” or “depression”) that the coders determined functionally corresponded to a DSM-5 entry or category. Whether a patient had a disorder that fell under a given DSM-5 chapter determined their membership in a given *broad* psychiatric disorder group for Aim 4. For example, a person who reported “personality disorder” or “dependent personality disorder” in addition to “anxiety” or “social anxiety disorder” would be classified as belonging to the personality disorders group as well as to the anxiety disorders group. The initial coders classified patient disorders according to the following DSM-5 categories: neurodevelopmental disorders, anxiety disorders, obsessive-compulsive and related disorders, trauma and stressor-related disorders, dissociative disorders, personality disorders, feeding and eating disorders, substance-related and addictive disorders, schizophrenia spectrum and other psychotic disorders, bipolar and related disorders, depressive disorders.

However, instead of the DSM categories “schizophrenia spectrum and other psychotic disorders,” “bipolar and related disorders,” and “depressive disorders,” we classified patients according to the categories “psychosis,” “bipolar mood features,” and “unipolar mood features.” Also, we added a new category, “unspecified mood features” (not to be confused with unspecified mood disorder (American Psychiatric Association, 2022)), to account for people who self-reported schizoaffective disorder but who did not self-report a mood specifier/disorder (e.g., manic or depressive symptoms). Inclusion in the unipolar mood features, bipolar mood features, and unspecified mood features categories were mutually exclusive. Thus, a person who said that they had both major depressive disorder and bipolar II disorder would be re-coded as having bipolar mood features but not unipolar mood features. This is in line with the DSM-5, according to which bipolar disorder supersedes major depressive disorder (American Psychiatric Association, 2022). We classified patients into the psychosis category if they self-reported psychosis, if they self-reported a disorder on the schizophrenia spectrum, and/or if they were coded as having psychosis for the “psychosis or not” stratification variable that was used for patient randomization (see Procedure). In effect, this new classification scheme treated psychosis as its own entity such that it counted toward one disorder for the number of psychiatric disorders variable (for Aims 1–3) regardless of whether it was part of a schizophrenia spectrum disorder, a mood disorder, or neither.

There were a few reasons for these steps. First, because of the wording of the psychiatric disorder question, it is possible that some people reported sequential or similar diagnoses, even in cases where one diagnosis was intended to replace another or when the diagnoses were mutually exclusive. If, for example, a person reported schizoaffective disorder and bipolar disorder with psychotic features, the ambiguity of their self-report would not pose a problem under this new scheme, as they would simply be coded for bipolar mood features and psychosis. In addition to clarifying instances of ambiguity, we determined that evaluating mood features and psychosis—rather than using corresponding DSM-5 categories—would be the most effective way to curtail deflation or inflation of comorbidity, particularly for Aims 1–3, which operationalized comorbidity as a quantitative, continuous variable. For example, the current coding scheme meant that a patient who reported only schizoaffective disorder and a patient who reported only schizophrenia and major depressive disorder would be given the same value for the number of psychiatric disorders variable (the former patient would be coded as having unspecified mood features and psychosis, and the latter would be coded as having unipolar mood features and psychosis). Finally, this coding scheme allowed for patients who had been diagnosed with mood disorders accompanied by psychotic features to be included in the psychosis group for Aim 4 (see below), despite not having disorders that classically fall on the schizophrenia spectrum.

Aim 4 relied on **broad psychiatric disorder groups** (see above). For this aim, we only analyzed the sample’s five most common groups, which included unipolar mood features, bipolar mood features, anxiety disorders, psychosis, and trauma and stressor-related disorders. We allowed for multiple disorders within the same *broad* diagnostic category in instances where one disorder did not supersede another in the DSM-5.

The **number of psychiatric disorders** variable (used for Aims 1–3) represented the sum of the number of disorders the patient reported, after data cleaning. We treated the number of psychiatric disorders as a continuous variable. Of the 539 patients who were enrolled in the parent trial, 50 were not coded for any DSM-5 disorders according to their self-reports. Because we did not consider these patients to be “true 0s” given that they were using the services of CMHCs, which treat patients with severe mental health challenges, these 50 patients were eliminated from all analyses in the current study. Thus, each model in Aims 1–3 used the available data from the remaining 489 patients. Aim 4 also drew from subsets of these 489 patients but only used the patients who fell into the five most common broad psychiatric categories. Many patients fell into more than one of these five categories. The sample sizes of these psychiatric disorder groups ranged from *N* = 118 to *N* = 292 (see Aim 4 in the Results). Supplementary Box 1 lays out details pertaining to how psychiatric disorder information was cleaned and re-coded.

### 2.4 Procedure

Providers and patients all consented prior to participation. During the parent trial, CMHCs and patients were randomized through a computerized randomization sequence. During the process of patient randomization, patients were stratified by presence of psychosis or not, presence of substance use or not, and age group (≥ 50 or not) (Callaway et al., 2023; Harvey et al., 2025).

Patients’ PROMIS-SD scores were collected at the screening stage to determine eligibility. Data was collected at multiple waves, though for the present study, only two time points—the pre-treatment/baseline and post-treatment scores—were used. In the UC-DT group, post-treatment measures reflected responses after usual care (only) and before delayed TSC. In the Immediate TSC group, post-treatment reflected post-TSC responses.

### 2.5 Data Analysis

Analyses were performed in RStudio using R version 4.4.1. Though our hypotheses were all directional in nature, we performed two-tailed analyses for all aims in order to detect possible significant effects that were in the opposite direction as the hypotheses. We calculated both uncorrected *p*-values and *p*-values corrected using the Benjamini-Hochberg procedure (Benjamini & Hochberg, 1995).

For Aim 1, we conducted five multiple regression analyses, each of which investigated a different outcome variable (psychiatric symptoms, sleep disturbance, sleep-related impairment, overall sleep health, or functional impairment) at pre-treatment, using weighted least squares. Each of the Aim 1 analyses included the same independent variable of interest (the number of psychiatric disorders) and included county and generation as covariates. We adjusted for county using nine dummy codes at the fixed level throughout.

For Aims 2–4, we performed intent-to-treat analyses, leveraging hierarchical linear modeling with random intercepts to account for multiple time points within patient. We tested five multilevel models for Aim 2 and five multilevel models for Aim 3. We conducted a total of 25 multilevel models for the Aim 4 analyses, examining each of the five outcome variables for each of the five most common psychiatric disorders in the sample. We used maximum likelihood estimation, which performs well in simulations of multilevel models with missing data up to 50% (Black et al., 2011), for Aims 2–4. While we applied transformations^6^ to address some instances of assumption violations throughout analyses, we also employed robust standard errors with bias-reduced linearization adjustments (Bell & McCaffrey, 2002; Pustejovsky & Tipton, 2018) for all models in Aims 2–4 in order to address possible issues with heteroskedasticity.

For Aim 2, we tested the three-way interaction between Time (dummy coded as 0 for pre-treatment and 1 for post-treatment), UC-DT vs. Immediate TSC (dummy-coded as 0 and 1, respectively), and the number of psychiatric disorders (treated as a continuous variable), where the interaction was modeled as a predictor of each outcome: psychiatric symptoms, sleep disturbance, sleep-related impairment, overall sleep health, or functional impairment. We adjusted for county and generation in addition to the stratifying variables (see Procedure): the dichotomous age variable, the dichotomous substance use disorder variable, and the dichotomous psychosis variable.

For Aim 3, we were primarily interested in the three-way interaction between Time, Adapted vs. Standard TSC condition (dummy-coded as 1 and 0, respectively), and the number of psychiatric disorders, where the interaction was modeled as a predictor of each outcome: psychiatric symptoms, sleep disturbance, sleep-related impairment, overall sleep health, or functional impairment. We initially adjusted for generation and county but ultimately dropped the county covariates for the Aim 3 models due to issues with multicollinearity. Corresponding Aim 3 models with and without county covariates did not significantly differ from one another in fit, with the exception of the SDS model (χ^2^(8) = 16.62, *p* = .034). Neither version of the Aim 3 SDS model produced a significant result for the interaction of interest.

For Aim 4, we tested the two-way interaction between Time and UC-DT vs. Immediate TSC condition, where the interaction was modeled as a predictor of each outcome: psychiatric symptoms, sleep disturbance, sleep-related impairment, overall sleep health, or functional impairment. We adjusted for county, generation, and the three stratifying variables (the same as for Aim 2).

## RESULTS

The final sample of 489 patients reported a mean of 2.29 (*SD* = 1.06) psychiatric disorders (median = 2.00, mode = 2.00) according to the diagnostic coding scheme described in Psychiatric Disorder Variables above.

Of the 489 patients, 36% were assigned male at birth, 63% were assigned female at birth, and 1% did not have data for this item. Additionally, 32% of patients were Hispanic and/or Latino, 67% were not, and 1% did not have data for this item. The racial distribution of patients was as follows: 8% American Indian or Alaskan Native, 2% Native Hawaiian or Pacific Islander, 51% White, 9% Asian, 12% Black or African American, 8% more than one race, 8% other, 2% missing. The sample was also socioeconomically and educationally diverse. Further details on the sociodemographic distributions of the full sample can be found in Harvey et al. (2024) (Harvey et al., 2025) (for generation 1) and in the forthcoming primary generation 2 paper.

**Figure 1:**
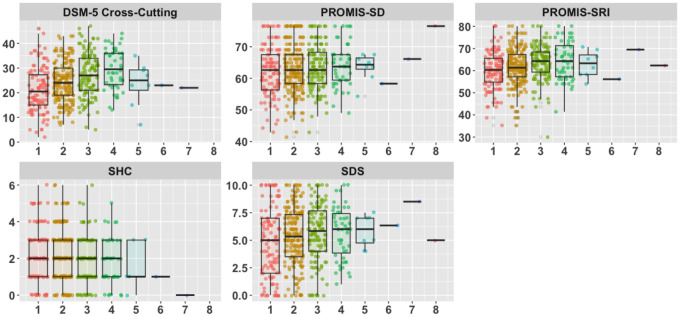
The number of psychiatric diagnoses plotted against patient baseline scores for each of the five main outcomes Note: DSM-5 Cross-Cutting = DSM-5 Cross-Cutting Measure; PROMIS-SD = Patient-Reported Outcomes Measurement Information System-Sleep Disturbance; PROMIS-SRI = Patient-Reported Outcomes Measurement Information System-Sleep Related Impairment; SHC = Sleep Health Composite; SDS = The mean of each patient’s available responses to the items on the Sheehan Disability Scale. Each point represents a different response. Each color corresponds to a different number of psychiatric diagnoses. Jitter is added to convey density. The overlaid box-and-whisker plots visualize the medians (the thick black lines), first quartiles (the lower bounds of the rectangles), and the third quartiles (the upper bounds of the rectangles) for each subgroup. Black lines without rectangles denote an equivalent median, first quartile, and third quartile. Points below the lower whiskers and above the upper whiskers are more than 1,5*the interquartile range below the 1st quartile and more than 1.5*the interquartile range above the 3rd quartile, respectively.

Figure 1 illustrates the distribution of the baseline scores for the five outcomes (in the present sample of 489 patients), stratified by the number of psychiatric disorders. Supplementary Tables 2 and 3 show the pre and post means and SDs for the five main outcomes in the Immediate TSC and UC-DT conditions and in the Adapted and Standard conditions, respectively. Supplementary Tables 4 and 5 display the pre and post means and SDs for the five main outcomes within the five most common broad psychiatric groups separately (see Aim 4) for UC-DT and Immediate TSC patients, respectively. For the remainder of the Results section, in instances where outcomes were transformed to address assumption violations, *p*-values associated with both the transformed and non-transformed versions of the outcome variable are reported.

### 3.1 Aim 1

Patients with a higher number of disorders had, on average, higher DSM-5 Cross-Cutting scores at baseline (*b* = 2.27, *SE* = 0.39, *p* < .001). The relationship between the number of psychiatric disorders and PROMIS-SD score was positive and significant prior to elimination of outliers (*b* = 0.71, *SE* = 0.31, *p* = .023). After a conservative elimination of outliers, the relationship was no longer significant (*b* = 0.57, *SE* = 0.32, *p* = .073). Patients with a higher number of disorders tended to have higher baseline PROMIS-SRI scores (*b* = 1.25, SE = 0.34, p < .001; *p*_transformed_ < .001), lower SHC scores (*b* = −0.20, *SE* = 0.06, *p* = .001; *p*_transformed_ = .004) (where higher scores indicate greater sleep health for the SHC), and higher mean SDS scores (*b* = 0.32, *SE* = 0.11, *p* = .005). After adjusting *p*-values using the Benjamini-Hochberg procedure, all Aim 1 results remained significant, excluding the version of the PROMIS-SD model that eliminated outliers.

### 3.2 Aims 2 and 3

Aims 2 and 3 evaluated whether the relationship between the number of psychiatric disorders and improvement on the five outcome measures depends on condition (Immediate TSC vs. UC-DT and Standard vs. Adapted, respectively). All Aim 2 and 3 analyses yielded non-significant interaction terms at the nominal (i.e., uncorrected) level (*p* > .05) for the primary coefficients of interest. Table 1 displays the resulting (non-transformed), fixed effects coefficients (*b*)s, after adjusting for covariates, along with the associated robust SEs and *p*-values.

### 3.3 Aim 4

For Aim 4, we evaluated whether patients in the most commonly reported *broad* psychiatric disorder groups—unipolar mood features, bipolar mood features, anxiety disorders, psychosis, and trauma and stressor-related disorders—showed more improvement in the Immediate TSC condition from pre to post when compared to the UC-DT group. Supplementary Table 6 displays the resulting (non-transformed), fixed effects coefficients (*b*)s, after partialing for covariates, along with the associated robust SE’s and p-values. Supplementary Table 7 displays the number of patients with each possible two-disorder combination of *all* of the broad disorder categories within the final sample of 489 patients. All results reported as significant below are significant at the corrected level except where otherwise noted.

#### 3.3.1 Unipolar mood features

Results for patients with self-reported unipolar mood features (*N* = 251) indicated greater improvement from pre to post in the Immediate TSC condition, relative to the UC-DT condition, across all five outcomes (DSM-5 Cross-Cutting Measure: *b* = −4.25, 95% CI: [−6.70, −1.80], *p* < .001; PROMIS-SD: *b* = −9.66, 95% CI: [−12.16, −7.15], *p* < .001; PROMIS-SRI: *b* = −8.74, 95% CI: [−11.67, −5.80], *p* < .001; *p*_transformed_ < .001; SHC: *b* = 1.72, 95% CI: [1.15, 2.28], *p* < .001; *p*_transformed_ < .001; SDS: *b* = −1.81, 95% CI: [−2.64, −0.97], *p* < .001).

#### 3.3.2 Bipolar mood features

Patients in the bipolar mood features group (*N* = 118) did not demonstrate greater improvement from pre to post in the Immediate condition, relative to the UC-DT condition, on the DSM-5 Cross-Cutting Measure (*b* = −1.10, 95% CI: [−4.71, 2.51], *p* = .549) but did with respect to PROMIS-SD (*b* = −11.82, 95% CI: [−16.82, −6.82], *p* < .001) and PROMIS-SRI (*b* = −8.92, 95% CI: [−13.12, −4.72], p < .001; *p*_transformed_ < .001). Prior to transformation, SHC was nominally significant in the positive direction (*b* = 0.81, 95% CI: [0.07, 1.54], *p* = .032), but neither the uncorrected p-value associated with the transformed SHC outcome (*p*_transformed_ = .057) nor the corrected *p*-value associated with the non-transformed SHC outcome (*p*_corrected_ = .082) was. SDS was significant at the nominal level (*b* = −1.52, 95% CI: [−3.02, −0.03], *p* = .046) but not at the corrected level (*p*_corrected_ = .068).

#### 3.3.3 Anxiety disorders

Patients in the anxiety disorders group (*N* = 292) indicated greater improvement from pre to post in the Immediate condition, relative to the UC-DT condition, across all five outcomes (DSM-5 Cross-Cutting Measure: *b* = −4.25, 95% CI: [−6.46, −2.04], *p* < .001; PROMIS-SD: *b* = −9.70, 95% CI: [−12.22, −7.17], *p* < .001; PROMIS-SRI: *b* = −8.08, 95% CI: [−10.68, −5.47], *p* < .001; *p*_transformed_ < .001; SHC: *b* = 1.43, 95% CI: [0.95, 1.92], *p* < .001; *p*_transformed_ < .001; SDS: *b* = −1.90, 95% CI: [−2.69, −1.11], *p* < .001).

#### 3.3.4 Psychosis

Patients in the psychosis group (*N* = 166) showed greater improvement from pre to post in the Immediate condition, relative to the UC-DT condition, for the DSM-5 Cross-Cutting Measure at the nominal level (*b* = −3.40, 95% CI: [−6.57, −0.24], *p* = .035) but not at the corrected level (*p*_corrected_ = .054), for PROMIS-SD (*b* = −7.84, 95% CI: [−11.34, −4.35], *p* < .001), for PROMIS-SRI (*b* = −7.62, 95% CI: [−11.36, −3.89], *p* < .001; *p*_transformed_ < .001), for SHC (*b* = 1.12, 95% CI: [0.53, 1.70], p < 0.001; *p*_transformed_ < .001), and for SDS (*b* = −1.95, 95% CI: [−3.10, −0.81], *p* < .001).

#### 3.3.5 Trauma and stressor-related disorders

Patients in the Trauma and stressor-related disorders group (*N* = 143) did not exhibit improvement from pre to post in the Immediate condition, relative to the UC-DT condition, for the DSM-5 Cross-Cutting Measure (*b* = −0.72, 95% CI: [−3.70, 2.25], *p* = .633). However, the Immediate condition did show greater improvement from pre to post with respect to the PROMIS-SD (*b* = −8.37, 95% CI: [−12.04, −4.70], *p* < .001), PROMIS-SRI (*b* = −7.84, 95% CI: [−11.52, −4.15], *p* < .001; *p*_transformed_ < .001), SHC (*b* = 1.29, 95% CI: [0.51, 2.07], *p* = .001; *p*_transformed_ < .001), and SDS (*b* = −1.32, 95% CI: [−2.50, −0.13], *p* = .029).

## DISCUSSION

In this secondary analysis, we evaluated outcomes from a community sample of patients with both SMI and sleep/circadian problems who received the Transdiagnostic Intervention for Sleep and Circadian Dysfunction (TSC). Patients were assigned to one of two forms of TSC (Standard or Adapted), which was delivered alongside usual care, with some patients receiving TSC immediately (Immediate TSC) and others receiving usual care alone followed by delayed treatment (UC-DT). The sample was clinically diverse: patients reported many different psychiatric disorders and combinations of psychiatric disorders. Additionally, most patients (76%) reported they had been diagnosed with at least two psychiatric disorders. This study’s overarching goal was to further knowledge on self-reported psychiatric disorders and comorbidities as predictors of symptom/impairment severity and treatment outcome within a sleep and circadian context.

Aim 1 tested the hypothesis that patients who self-reported more psychiatric disorders had more psychiatric symptoms, more sleep disturbance, more sleep-related impairment, poorer overall sleep health, and greater functional impairment at baseline. This hypothesis was generally supported, though with slightly less evidence for sleep disturbance. These findings largely parallel those from Sarfan et al. (2021) (Sarfan et al., 2021), which were based on a similar community sample. After adjusting for diagnostic threshold, Sarfan et al. (2021) found that having multiple *sleep/circadian* problems, compared to one sleep/circadian problem, was usually associated with greater baseline sleep-related impairment, worst overall sleep health, and greater functional impairment. Sarfan *et al*. did not find a statistically significant relationship between number of sleep and circadian problems and baseline sleep disturbance score—though the relationship trended toward significance (*p* = .07). Meanwhile, in the current study, the significance of the relationship between number of *psychiatric disorders* and baseline sleep disturbance score depended on whether outliers were removed. More broadly, our findings add to the existing literature suggesting that number of psychiatric disorders is positively correlated with the severity of psychiatric symptoms/disorder(s) (Brown et al., 1995; Kessler et al., 2005), as well as with indices of distress, impairment, and quality of life (Angst et al., 2002).

Aim 2 tested whether the benefits of TSC were greater for people with fewer psychiatric disorders. Aim 3 tested whether the number of psychiatric disorders moderated the extent to which patients benefited from one version of TSC over the other. The Aim 2 and 3 results were all non-significant, with most yielding high *p*-values. In other words, the Aim 2 results presented no statistically significant evidence for an association between number of psychiatric disorders and TSC treatment outcome; similarly, the Aim 3 results presented no statistically significant evidence that number of psychiatric disorders was associated with effectiveness of Adapted TSC relative to Standard TSC. Past research on the relationship between psychiatric comorbidity and treatment effect/outcome has yielded variable results. The direction of this relationship may depend on factors such as the index condition(s) in question, whether pharmacological history is considered, the ways in which improvement or recovery is measured, and whether post-treatment or follow-up outcomes are being considered (Brown et al., 1995; Huang et al., 2020; Liber et al., 2010; Olatunji et al., 2010). Interestingly, Sarfan et al. (2021) did not find statistically significant evidence that patients’ improvement on outcomes over the course of TSC was associated with number of (*sleep and circadian*) problems (Sarfan et al., 2021).

Aim 4 evaluated whether, when considered independently, patients in the five most common broad psychiatric disorder groups—unipolar mood features, bipolar mood features, anxiety disorders, psychosis, and trauma and stressor-related disorders—benefited more in the Immediate TSC group than in the UC-DT condition. Across the Aim 4 analyses, all *significant* results favored the Immediate TSC group over the UC-DT group. All five groups demonstrated significantly more improvement in the Immediate TSC group than in the UC-DT group on sleep-related disturbance and impairment. The bipolar, psychosis, and trauma groups showed a combination of significant results and non-significant results—all in the expected direction—for psychiatric symptoms, overall sleep health, and functional impairment. These findings are unsurprising, as prior analyses found that, in generation 1 (Harvey et al., 2025) and generation 2 (on submission) of the current sample, sleep disturbance and sleep-related impairment were associated with the largest standardized treatment effects of all five outcomes. The non-significant Aim 4 results may be related to the latter three psychiatric disorder groups (psychosis, trauma, and bipolar) having the smallest sample sizes. While degree of patient improvement varied across measures, the Aim 4 results are generally encouraging. The results suggest that a transdiagnostic sleep treatment is a particularly valuable approach to treating populations with SMI. This is consistent with the accruing evidence that sleep is an important mechanistic contributor to the course of psychiatric disorders. For example, in schizophrenia, sleep and circadian processes have been implicated in worsening of delusions, hallucinations, and sometimes other symptoms (Freeman et al., 2020). Sleep has been associated with processing of traumatic events, and poor sleep is correlated with the development of PTSD in response to traumatic events (Freeman et al., 2020; Harvey et al., 2003). Additionally, sleep disturbance is a common prodrome of mania in bipolar disorder (Jackson et al., 2003). Because numerous findings point to sleep-related treatment as an indirect path to alleviating symptoms across the psychopathology spectrum (Freeman et al., 2020; Harvey et al., 2021, 2025; Rumble et al., 2015; Yau et al., 2024), research should continue to evaluate sleep and circadian problems as an important transdiagnostic target.

These findings need to be interpreted in light of several limitations. First, we cannot rule out the possibility that the analyses for Aims 2 and 3 were underpowered to detect true effects, particularly given that detecting three-way interactions requires a substantially larger sample size than detecting equivalent two-way interactions (Heo & Leon, 2010). Next, since a large proportion of patients had missing provider-reported psychiatric diagnosis data and for other reasons highlighted throughout this paper, the current study relied on patients’ self-report of psychiatric diagnoses they had received. Past research on the accuracy of retrospective psychiatric self-reports is mixed: in some research and for certain disorders, patients tend to recall their history well, while in other cases, recall is poor (Patten et al., 2012; Prusoff et al., 1988; Simon & VonKorff, 1995; Takayanagi et al., 2014). However, since patients in the present community sample were all receiving treatment at a CMHC for an SMI, their recall of their diagnostic history may have been better than average. Finally, as detailed in the Methods, the first item on the Sheehan Disability Scale (SDS) had an unusually high level of missingness. Since higher scores indicate greater impairment, those who had missing item 1 scores because their high level of impairment made them unable to work or attend school likely would have appeared substantially less impaired than they were had their SDS scores been summed. Our decision to use the mean across patients’ non-missing SDS items was less likely to lead to artificial deflation in patients who did not respond to the first item.

## CONCLUSIONS

The analyses in this paper showed that, on average, community sample patients with a higher number of self-reported psychiatric disorders began the study with more psychiatric symptoms, more sleep disturbance, more sleep-related impairment, poorer overall sleep health, and greater overall functional impairment. We did not find an association between the number of psychiatric disorders and treatment effects—across either the UC-DT vs. Immediate TSC or Adapted vs. Standard analyses. Thus, while patients with more psychiatric disorders began treatment with more symptoms, sleep/circadian problems, and overall impairment, there was no evidence that they showed fewer absolute gains over the course of TSC or that they were more likely to benefit from either version of the treatment over the other. When considering the sample’s most common psychiatric disorder groups independently, the Immediate TSC group demonstrated more improvement in outcomes than did the UC-DT group for most outcome x disorder group combinations. While prior research has demonstrated TSC’s benefits in populations with SMI (Harvey et al., 2021, 2025; Yau et al., 2024), the current paper extends the scope of knowledge on TSC by further establishing its connection to comorbidity.

## Supplementary Material

Supplementary Files

This is a list of supplementary files associated with this preprint. Click to download.

- SupplementaryMaterialAPMHMHSR.pdf

## Figures and Tables

**Table 1:** Fixed effects coefficients (*bs*), standard errors, and *p*-values associated with Aims 2 and 3, after adjusting for covariates

Outcome	*TSC x Time x Disorders*	*SE*	*p*	*Adap/Stand x Time x Disorders*	*SE*	*p*
DSM-5	0.940	0.791	.235	−0.537	0.844	.524
PROMIS-SD	0.694	1.053	.510	−0.283	1.210	.815
PROMIS-SRI	0.667	0.944	.480	1.552	1.160	.181
SHC	0.134	0.188	.478	0.023	0.234	.922
SDS	−0.016	0.303	.959	0.093	0.364	.798

Note: For Aim 2, the coefficient is the three-way interaction between Time, UC-DT vs. Immediate TSC condition, and the number of psychiatric disorders; for Aim 3, the coefficient is the three-way interaction between Time, Adapted vs. Standard condition, and the number of psychiatric disorders. Non-transformed coefficients are included here for ease of interpretation. For Aim 3, the above output is based on the models that do not adjust for county. Across aims, the listed SEs and *p*-values are based on robust estimation; the *p*-values listed are not corrected for multiple testing.

## Data Availability

All research materials, data, and analysis code are available from the authors upon request. Raw data for most outcomes have been uploaded onto the National Data Archive. A treatment manual can be obtained upon request from the authors.

## Footnotes

1. Some papers use the term “multimorbidity,” specifically when describing the relationship between multiple disorders without a focal condition, as distinct from comorbidity (i.e., liver cancer with comorbid conditions). “Multimorbidity” is often used in the epidemiological literature and in studies that (additionally or exclusively) consider medical, non-psychiatric conditions.

2. This approach was taken in some cases when the patient and provider were uncertain about the SMI diagnosis. However, many patients had both participant and provider diagnosis data, while a minority of patients had neither.

3. Providers were trained to deliver Adapted TSC in 4 sessions and Standard TSC in 8 sessions, though in actuality, treatment was often delivered on an alternate timeline.

4. As the project evolved, we relabeled the first three core module numbers in the Adapted condition “1, 2, and 3,” in contrast to the corresponding Standard labels “1a, 1b, and 1c” to help the providers follow that these were separate modules to be delivered in different sessions (see Supplementary Table 1).

5. The DSM-5-TR is a Text Revision of the DSM-5 in which prolonged grief disorder, unspecified mood disorder, and stimulant-induced mild neurocognitive disorder first appeared as official entities. The DSM-5-TR also includes amendments and clarifications relating to symptom codes, specifiers, and terminology, as well as updates to information about risk factors, prevalence, culture-/sex-/gender-related features, etc. (American Psychiatric Association, 2022; First et al., 2022; *Updates to DSM-5-TR Criteria and Text*, n.d.).

6. Throughout Aim 1–4, we applied square root transformations or Box Cox transformations—depending on the analysis—to some outcomes.
